# ASSOCIATION BETWEEN BALANCED DIET AND FRAILTY IN JAPANESE OLDER ADULTS: THE MODERATING ROLE OF SOCIAL PARTICIPATION

**DOI:** 10.1093/geroni/igad104.1842

**Published:** 2023-12-21

**Authors:** Yaya Li, Yuya Akagi, Natsumi Fujiwara, Hiroko Yoshida, Michiko Kido, Mai Kabayama

**Affiliations:** Osaka University, Suita, Osaka, Japan; Osaka University, Suita, Osaka, Japan; Osaka University, Suita, Osaka, Japan; Osaka University, Suita, Osaka, Japan; Osaka University, Suita, Osaka, Japan; Osaka University, Suita, Osaka, Japan

## Abstract

The association between diet and frailty in older adults may be related to social factors. This study aimed to investigate whether there is an interaction effect between balanced diet and social participation on frailty in older Japanese. Balanced diet was defined as the consumption of more than one meal per day that contained carbohydrates, protein, and fiber. Data were collected from 4,832 community-dwelling older adults aged 65-89 years through self-reported questionnaires (response rate 49.0%). Frailty was measured using the Kihon Checklist developed by the Ministry of Health, Labour, and Welfare. We stratified participants by age (65-74 and 75-89 years) and conducted logistic regression models adjusted for economic status, gender, marital status, living alone, disease, and physical activity. The interaction between dietary diversity and social participation in frailty was also assessed. Results showed that in both age groups, consuming balanced diet was associated with a lower risk of frailty (OR=0.56, 95%CI=0.40–0.79). Lack of social participation was found to be a risk factor for frailty (OR=1.51, 95%CI=1.03–2.21). Furthermore, the interaction between balanced diet and social participation revealed that a combination of lack of social participation and balanced diet was also associated with a lower frailty risk in the 75-89 age group (OR=0.63, 95%CI=0.40–0.99). These findings suggest that balanced diet consumed at least once a day may be beneficial for preventing frailty for older adults, however, social participation should also be taken into consideration for those aged 75-89. This study has some limitations as a cross-sectional study. Longitudinal research is needed.

